
JOURNAL INFORMATION
==============================
NLM Title Abbreviation: ScientificWorldJournal
Journal ID: ScientificWorldJournal
NLM Journal ID: 101131163
Journal ID: TSWJ

The Scientific World Journal

ISSN: 2356-6140
EISSN: 1537-744X
Publisher: Wiley

ARTICLE INFORMATION
==============================
PMCID: PMC5956393
PMID: 15632985
DOI: 10.1100/tsw.2004.187
Article ID: 258064
Article version: 1
Subjects: Case Study

Paraneoplastic Hypercalcaemia Associated with TCC of Bladder

Patel A. B. 1 *
Wilson L. 2
Blick C. 2
Meffan P. 2
1 Institute of Urology and Nephrology, UCL Medical School, Charles Bell House, 67 Riding House Street, London, UK
2 Department of Urology, East Surrey Hospital, Redhill, Surrey and Sussex NHS Trust, U.K
*A. B. Patel: amitpat99@aol.comAcademic Editor: Anthony Atala

Publication date: 2004
Electronic publication date: 2004 Dec 10
Volume: 4
First page: 1069
Last page: 1070
Received 2004 Jul 14; Revised 2004 Sep 28; Accepted 2004 Oct 5
Copyright: Copyright © 2004 A. B. Patel et al.
Copyright year: 2004
License: This is an open access article distributed under the Creative Commons Attribution License, which permits unrestricted use, distribution, and reproduction in any medium, provided the original work is properly cited.
License URL: https://creativecommons.org/licenses/by/3.0/

Keywords: TCC, hypercalcaemia, paraneoplastic syndrome

==============================
Case Study
*TheScientificWorldJOURNAL* (2004) 4, 1069–1070
ISSN 1537-744X; DOI 10.1100/tsw.2004.187

### Paraneoplastic Hypercalcaemia Associated with TCC of Bladder

A.B. Patel[1,]*, L. Wilson[2], C. Blick[2], and P. Meffan[2]

[1]*Institute of Urology and Nephrology, UCL Medical School, Charles Bell House, 67 Riding House Street, London;* [2]*Department of Urology, East Surrey Hospital, Redhill, Surrey and Sussex NHS Trust, U.K.*

**E-mail**: amitpat99@aol.com

*Received July 14, 2004; Revised September 28, 2004; Accepted October 5, 2004; Published December 10, 2004*

**KEYWORDS**: TCC, hypercalcaemia,paraneoplastic syndrome

**DOMAIN:** urology

Hypercalcaemia associated with transitional carcinoma of the bladder is rare in the absence of metastatic disease. We report a case of paraneoplastic hypercalcaemia associated with a high-grade, organ-confined bladder carcinoma, which resolved upon surgical resection of the tumour.

### CASE REPORT

A 58-year-old woman presented with a 3-week history of weight loss, general malaise, and constipation as well as two episodes of macroscopic haematuria. Laboratory testing on admission to hospital showed marked hypercalcaemia (3.66, normal 2.15–2.60) associated with a PTH level of 1.3 (normal 0.5–5.5) and acute renal failure (BUN 37.5, creatinine 280). Ultrasound examination showed moderate right hydroureteronephrosis associated with a large, lobulated echogenic mass in the bladder. Immediate treatment involved intravenous colloid infusion, intravenous furosemide, and intravenous pamidronate in order to treat the hypercalcaemia.

Subsequent transurethral bladder resection revealed grade 3 pT2 transitional cell carcinoma (TCC). Staging computerised tomography and bone scan showed no evidence of local or distant metastatic disease. The patient then underwent radical cystectomy and ileal conduit formation. Pathological examination confirmed urothelial cell bladder carcinoma grade 3 pT4a.

Postsurgery calcium levels returned to normal limits.

### DISCUSSION

A paraneoplastic syndrome is defined as a constellation of symptoms that occur due to substances produced by a tumour. While this can occur in up to 20% of patients with renal cell carcinoma, it is a rare phenomenon in transitional cell carcinoma. It is evident from the available literature that TCC associated with paraneoplastic syndrome is a rare entity with only eight such cases being described since 1979 (Pubmed database) and most of these are high grade and aggressive in nature. Interestingly, most of the cases of hypercalcaemia are associated with upper tract TCC rather than bladder tumour.

*Corresponding author: Tel: 07803 505 515/ Fax: 01737 231 803
©2004 with author.

Parathyroid hormone-related peptide (PTH-rp) is the most common cause of humoral hypercalcaemia of malignancy[1,2]. PTH-rp is a paracrine/autocrine factor produced in almost every cell type in the body at some point, whether in childhood development or normal adult life. The peptide is critical for normal life, but is also the agent responsible for humeral hypercalcaemia of malignancy. This protein is homologous to parathyroid hormone (8 of the initial 13 amino acids in each protein are identical) and this structural similarity results in similar biologic actions. Both produce humeral hypercalcaemia by increasing bone resorption as well as increasing the renal absorption of calcium and hypophosphataemia via a phosphaturic effect on the kidney. Overall, 80% of hypercalcaemic cancer patients will have elevated levels of PTH-rp.

Less commonly, Vit 1,25 [$OH_2$] D3 has been implicated in the pathogenesis of paraneoplastic syndrome by acting to increase both $Ca^{2+}$ and phosphate reabsorption from the gut and kidney as well as by stimulating osteoblastic bone resorption. More recently, G-CSF (granulocyte colony-stimulating factor) producing bladder cancer has been described in the literature resulting in leucocytosis in association with hypercalcaemia[3]. It is thought to act by stimulating granulocyte proliferation and differentiation, augmenting autocrine growth resulting in aggressive poor prognostic tumours. Whether G-CSF has a synergistic role with PTH-rp inducing hypercalcaemia has yet to be elucidated.

Increased prostaglandin production by the tumour causing hypercalcaemia has also been postulated and in rare cases inhibitors of prostaglandin synthesis have corrected hypercalemia.

The case described fulfils the criteria for a paraneoplastic syndrome. The patient had symptomatic hypercalcaemia with proven localised bladder TCC without evidence of bony metastasis. The hypercalcaemia subsequently resolved after removal of the primary tumour. Unfortunately PTH-rp was not able to be measured pre- or postoperatively as it was not available in regional or supraregional hospital biochemistry laboratories. While the exact agent causing the hypercalcaemia cannot be defined, there is little doubt that this patient had a paraneoplastic syndrome.

Similar to renal cell carcinoma, TCC associated with paraneoplastic hypercalcaemia tends to be high grade and aggressive in nature with a poor prognosis[4]. Of the eight cases reported in the literature, four patients died and one had recurrence within 1 year.

In patients such as this, serum calcium may be of benefit as part of surveillance after radical treatment.

### REFERENCES

1. Becq-Giraudon, B., Marechaud, R., Pouget Abadie, J.F., and Sudre, Y. (1979) Paraneoplastic hypercalcaemia in a case of carcinoma of the bladder. Demonstration in the tumour of a substance with parathormone activity. *Nouv. Presse Med*. **8(16),** 1333–1335
2. Michel, F., Gattegno, B., Meyrier, A., Paillard, F., Roland, J., Scetbon, V., and Thibault, P. (1984) Paraneoplastic hypercalcaemia associated with bladder carcinoma. *J. Urol*. **131(4),** 753–755.
3. Kiyoshi, H., Tadaichi, K., Teruaki, O., and Hiroshi, M. (2002) Bladder tumor producing granulocyte colony-stimulating factor and parathyroid hormone related protein. *J. Urol*. **167,** 2130.
4. Chaudhary, U.B., Milling, D.L., and Bissada, N.K. (2004) Transitional cell carcinoma of the bladder producing parathyroid hormone-related protein. *Can. J. Urol*. **11(1),** 2136–2138.

**This article should be referenced as follows:**

Patel, A.B., Wilson, L., Blick, C., and Meffan, P. (2004) Paraneoplastic hypercalcaemia associated with TCC of bladder. *TheScientificWorldJOURNAL* **4**, 1069–1070.

**Handling Editor:**

Anthony Atala, Principal Editor for *Urology* — a domain of *TheScientificWorldJOURNAL*.
